# Type of surgical treatment and recurrence of oral leukoplakia: A retrospective clinical study

**DOI:** 10.4317/medoral.21645

**Published:** 2017-08-16

**Authors:** Luís Monteiro, Cinzia Barbieri, Saman Warnakulasuriya, Marco Martins, Filomena Salazar, José-Júlio Pacheco, Paolo Vescovi, Marco Meleti

**Affiliations:** 1 Medicine and Oral Surgery Department, Cancer Research Group, Institute of Research and Advanced Training in Health Sciences and Technologies (IINFACTS), University Institute of Health Sciences (IUCS), CESPU, Portugal; 2 Stomatology and Dental Medicine Department, Valongo Nossa Senhora da Conceição Hospital, Portugal; 3 Unit of Oral Medicine and Laser Surgery; Department of Medicine and Surgery, University of Parma, Italy; 4 Oral Medicine, King’s College London, the WHO Collaborating Centre for Oral Cancer, London, United Kingdom

## Abstract

**Background:**

Oral leukoplakia (OL) is the most typical potentially malignant disorder of the oral mucosa. We aimed to evaluate the clinical outcome of oral leukoplakia treated with several types of lasers and with the use of quantic molecular resonance (QMR) lancet, in terms of recurrence rate.

**Material and Methods:**

Eighty-seven previously untreated OL (52 occurring in females and 35 in males, mean age of 59.4 ± 13.9 years) were consecutively submitted to surgical treatment at University Hospital of Parma, Italy, and Hospital de Valongo, Portugal, (1999 to 2012). Interventions were subclassified into 5 groups according to the instrument used for the surgical removal of OL (cold blade – 17; Nd:YAG 1064nm laser – 14; Er:YAG 2940nm laser - 33; CO2 10600nm laser – 15; and QMR scalpel – 8). The mean follow-up period after treatment was 21.6 months (range 1-151 months). The outcome of treatment was scored through the same clinical protocol in the two participating units. Statistical analysis were carried by univariate analysis using chi-square test (or Pearson´s test when appropriate).

**Results:**

Recurrences were observed in 24 cases of OL (27.6%). Malignant transformation occurred in one patient (1.1%) after a period of 35 months. Statistical comparison of the 5 surgical treatment modalities showed no differences in clinical outcomes nor in the recurrence rate of OL. However, when Er:YAG laser group was compared with traditional scalpel, a significantly better outcome in cases treated with Er:YAG laser (*P* = 0.015) was highlighted.

**Conclusions:**

Our results suggests that Er:YAG laser could be a promising option for the treatment of OL.

** Key words:**Oral leukoplakia; Potentially malignant disorders; Er:YAG Laser; CO2 Laser; Nd:YAG Laser; Quantic molecular resonance scalpel; malignant transformation rate.

## Introduction

Oral leukoplakia (OL) is the most typical potentially malignant disorder of the oral mucosa, the others being oral lichen planus, erythroplakia and submucous fibrosis (1). In 2005, a workshop coordinated by the World Health Organization (WHO) Collaborating Centre for Oral Cancer and Precancer defined oral leukoplakia as “a white plaque of questionable risk having excluded (other) known diseases or disorders that carry no increased risk for cancer“ (1). The reported worldwide prevalence of OL for all ages is approximately 1-2 % (2). OL is more prevalent after the age of 30, with a gender variation in different societies, ranging from a male predominance in India, to an equal distribution in the western world (3). Tobacco is the most common etiologic factor although OL can occur in non-consumers of tobacco (4). In addition to tobacco consumption, alcohol seems to be an independent risk factor (5). Human papillomavirus infection has been associated with OL although with conflicting data (6).

Two clinical presentations of OL are recognized, the homogeneous and the non-homogeneous one. Homogeneous OL are uniform flat, thin, and white in color. They are usually asymptomatic and the risk of transformation is relatively low. Non-homogeneous OL include speckled or erythroleukoplakia (mixed red and white, but retaining predominantly the white character), nodular subtype (small polypoid outgrowths, rounded red or white excrescences), and verrucous subtype (wrinkled or corrugated surface) (1). When OL is widespread in the oral cavity this condition can be referred to as proliferative verrucous leukoplakia (PVL) (1). OLs may present with a spectrum of histopathological changes, ranging from simple hyperkeratosis to grades of dysplasia.

The reported malignant cumulative transformation rates of OL range between 0.1 to 36.4% (7) with an annual malignant transformation rate around 2-3% (4). Taking in account the potential malignancy of OL, the elimination of this condition is advisable as an attempt to avoid malignant transformation (8). Chemoprevention of OL using several medical interventions (Vitamin A, retinoids, beta-carotene, bleomicin) or mouthwash therapy containing an attenuated adenovirus has not been successful in prevention of transformation or complete elimination of OL. Several surgical modalities for the treatment of OL are reported including conventional cold scalpel excision, cryosurgery, laser excision or laser vaporization, and photodynamic therapy (9) but their different outcomes have not been ascertained.

The use of laser in the treatment of OL has been proposed because of several advantages inherent to its own biological properties including the potential haemostatic effect with a bloodless field, reduced postoperative pain, swelling, edema and infection (9). Moreover, the possibility of obtaining a second intention healing allows the treatment of large areas affected or localized in disfavorable oral sub-sites such as the floor of the mouth or palate, that are a challenge to intervene with the scalpel. Several lasers has been used to treat OL including CO2 laser, Nd:YAG laser, and KTP laser (10-12). Nevertheless, the different treatment modalities used have reported recurrence rates of OL in a wide range from 2 to 40% with an annual recurrence rate from 5 to 10% (4,13,14).

The objective of this study was to evaluate the clinical outcome of oral leukoplakia treated with different types of lasers, the quantic molecular resonance (QMR) lancet, and the cold scalpel in terms of resolution, prevention of recurrences and malignant transformation.

## Material and Methods

The cases of OL included in this multi-centre retrospective study were selected from the databases of the Center of Oral Pathology, Oral Medicine and Laser Surgery of the University Hospital of Parma, Italy (from 1999 to 2012) and of the Oral Medicine and Stomatology Department of Hospital de Valongo (Nossa Senhora da Conceição de Valongo / CESPU), in Oporto, Portugal (between 2007 and 2012). Institutional approval from ethics committee was obtained. Informed consent to treatment and use of data was obtained by each patient. The study was performed in accordance with the Declaration of Helsinki.

Data of 105 patients with OL were retrieved and reviewed included medical history charts, surgical reports follow up, according to the classification systems reported below. We included all consecutive previously untreated OLs located on the lip mucosa (C00.3-C00.5) or oral cavity (ICD-10: C01-06) with clinical and histological confirmation (with degree of diagnostic certainty C3 or C4, (according to the classification of van der Waal *et al.*) (4), and with data on follow-up. Patients with history of previous oral carcinomas, chemotherapy or radiotherapy, or cases with missing clinical or pathological data were excluded. Cases dropped off regarded 28 patients without histological confirmation and 17 cases without clinical data or follow-up. Sixty patients were eventually selected for the final evaluation and subclassified as follows: 36 (60%) females and 24 (40%) males with an average age at the time of initial diagnosis of 59.4 ± 13.9 years (range: 35-89 years). Detailed data retrieved included gender, age at the first visit, distribution of the oral lesions (single, multiple or multifocal), site of the lesion, macroscopic aspect (clinical type), tobacco and/or alcohol consumption, surgical treatment modality used for their intervention, histopathologic features including the assessment of dysplasia grade and follow-up information. These clinical-pathological characteristics are listed in  Table 1.

**Table 1:**
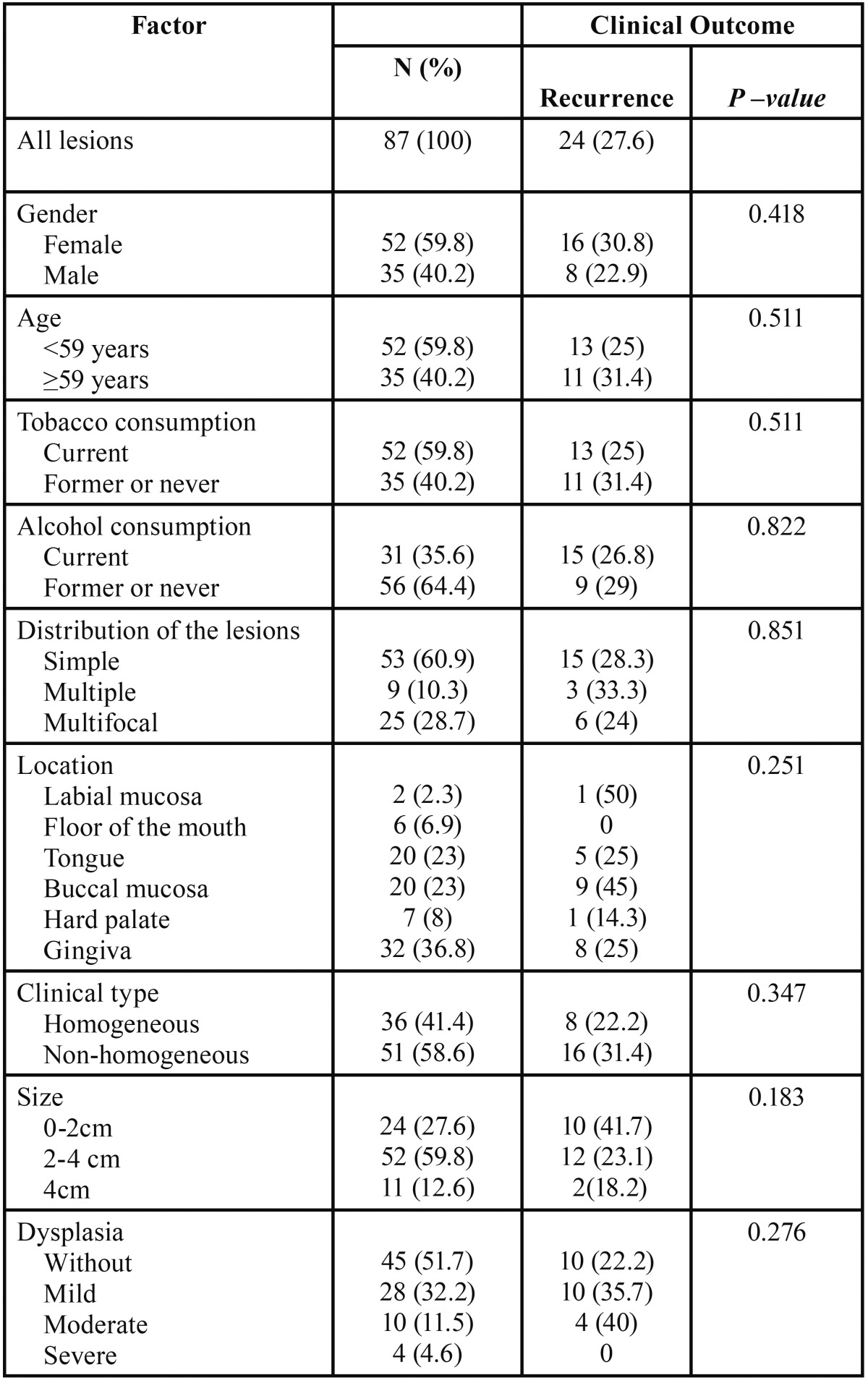
Analysis of the clinical-pathological characteristics of the 87 lesions with the presence of recurrence.

The clinical type of OL was classified according to Warnakulasuryia *et al.* (1), as homogeneous or non-homogeneous (with erythroleukoplakia, nodular and verrucous type including proliferative verrucous leukoplakia).

The presence of dysplasia was recorded according the WHO classification (2005) and graded as absent, mild, moderate, or severe dysplasia. All patients were given tobacco cessation advice prior to any surgical intervention.

According to treatment of lesions, patients were subclassified into five groups: 1- excision with traditional scalpel; 2- Excision with a quantic molecular resonance (QMR); 3- excision with Nd:YAG laser; 4- excision/vaporization with Er:YAG laser; and 5- vaporization with CO2 laser. In the present evaluation each OL lesion was considered individually with reference to the type of treatment.

The surgical protocol followed for all patients was similar in the two institutions: infiltration of an anaesthetic solution (2% lidocaine with 1:100,000 epinephrine) at 1 cm distance from the lesion; excision with resection margins including clinically healthy tissue at 3 mm from the lesion and extension in depth of 3 mm (when possible) below the lesion clinically visible. In the case of laser vaporization we started by marking a laser line on clinically healthy tissue extended by 3 mm of the margin of the lesion. Then we made a series of horizontal and vertical passes to reach the connective tissue (extension in depth of 3 mm, when possible, below the lesion clinically visible). In all vaporization cases a diagnostic incisional biopsy was performed and the histopathological diagnosis was obtained before surgical treatment. In case of necessity, non-absorbable suture wires 4.0 were used to obtain first intention healing of surgical wound in some cases. In the remaining cases the intervention site was left to heal by second intention after haemostasis carried out through mechanical compression, laser or QMR scalpel.

The characteristics and physical parameters of the instruments used in each treatment modality were as follows: traditional excisional surgery (cold scalpel) was performed with a Bard-Parker scalpel blade number 15 with a number 3 handle; Nd:YAG laser (wavelength of 1064nm - FOTONA®, Fidelis Plus, Slovenia) was used with a 320-μm fiber, with an output power of 3.5W and frequency of 70Hz (Power Density: 4375 W/cm2; Fluence: 62.5 J/cm2); Er:YAG laser (wavelength of 2940nm - FOTONA®, Fidelis Plus, Slovenia) was used with “very short pulse” (VSP), energy of 250mJ and frequency of 25 Hz (Power Density: 1250 W/cm2; Fluence: 50 J/cm2); CO2 laser (wavelength of 10600nm - DEKA® Smart US 20D, Firenze, Italy) was used with angulated mirror handpiece, defocalized and continues mode, 2-mm spot, with a power of 5W (power density of 159.2W/cm2 and fluence of 159.2 J/cm2); and QMR scalpel (Bladion® - TELEA, Quinto Vicentino, Vicenza, Italy) was used with the thin straight electrode (diameter: 0.15-mm). Safety precautions for protecting the operator, patient, and assistant were followed. Postoperatively 0.12% chlorhexidine mouthwash, non-steroidal anti-inflammatory drugs and hyaluronic acid with amino acids gel were prescribed to all patients to avoid infections and promote normal re-epithelialization of the site.

The patients were reviewed by the operator at 1 week, 4 weeks and every 6 months for the whole follow-up.

The clinical outcome was analysed according to the presence of recurrence or the presence of malignant transformation. Recurrence was defined as the presence of a white lesion within the borders of the treated area (in size more than 20% of previous lesion), irrespective of time interval. The malignant transformation was defined as a malignant tumour arising within the site of a treated OL. The overall rate of malignant transformation was calculated. The follow-up period (expressed in months) for each lesion was considered from the date of surgery until the last visit or up to the appearance of a recurrence.

-Statistics

Statistical analysis were carried out using IBM SPSS Statistics® version 21.0 software (IBM Corporation, NY, US). The results were expressed in absolute and relative frequencies. Any associations between socio-demographic, clinic-pathological variables versus outcome results were analyzed by univariate analysis using chi-square test (or Pearson´s test when appropriate). Differences were considered statistically significant at *P*<0.05.

## Results

There were 49 patients (81.7%) with a single lesion, 3 (5%) patients with multiple lesions, and 8 (13%) with multifocal lesions yielding a total of 87 OL lesions submitted to treatment. The most common location of OL was the gingival mucosa (including mucobuccal fold) (n=32; 36.8%), followed by buccal mucosa (n=20; 23%), tongue (n=20; 23%), palate (n=7; 8%), floor of the mouth (n=6; 6.9%), and labial mucosa (n=2; 2.3%). There were 36 (41.4%) homogeneous OL and 51 (58.6%) non-homogeneous OL constituted by 4 (4.6%) speckled OL, 1(1%) nodular OL, 39(44.8%) vecurrous OL including 18 PVL, and 7 (8%) non-homogeneous (not-otherwise specified) OL. Further clinical-pathological characteristics of these lesions are presented in the  Table 1.

The mean follow-up period was 21.6 months (range 1-151 months).

We observed treatment success in 63 (72.4%) lesions and recurrence (treatment failure) in 24 lesions (27.6%). None of the clinical and pathological variables taken into account were significantly associated with the clinical outcome ( Table 1).

Out of 87 OL treated with surgical methods, 17 (19.5%) were excised with a traditional scalpel, 8 (9.2%) with QMR scalpel, 14 (16.1%) with Nd:YAG laser, 33 (37.9%) were excised and/or vaporized with Er:YAG laser, and 15 (17.2%) vaporized with CO2 laser.

There were 8 (47%) failures in the traditional scalpel group, 2 (25%) failures in QMR group, 5 (35.7%) failures in the lesions submitted to Nd:YAG laser surgery, 5 (15.2%) failures in Er:YAG laser group, and 4 (26.7%) failures in the lesions vaporized with CO2 laser. In the overall analysis for the presence of recurrences following all treatment modalities no significant differences were detected (*P* = 0.179) ( Table 2). However, the comparison of each instrument used for leukoplakia treatment against tradi-tional scalpel revealed a statistical difference between Er:YAG laser group and cold knife group showing a significantly better outcome for the lesions treated with Er:YAG laser compared with the traditional scalpel (*P* = 0.015) ( Table 2).

**Table 2:**
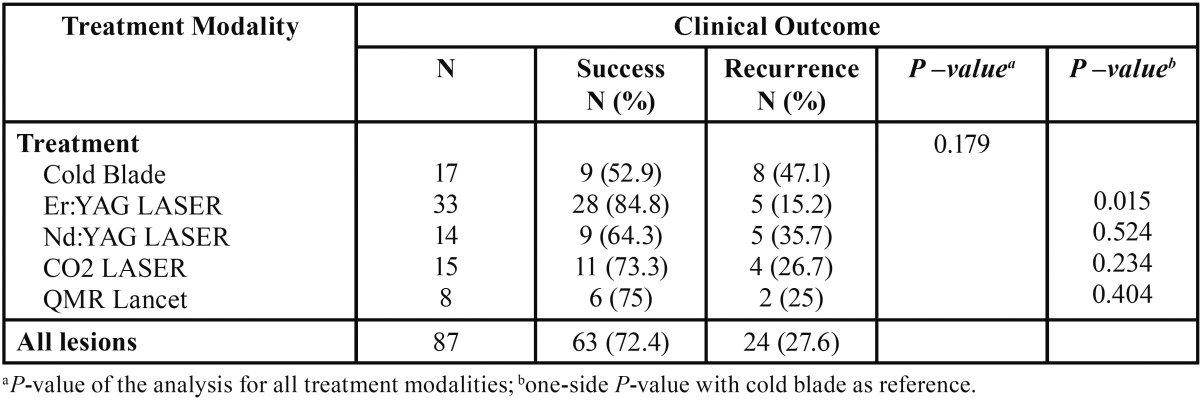
Analysis of the clinical outcome of the 87 lesions by treatment modality.

One lesion (1.1%) in a female patient (1.7%) underwent malignant transformation thirty-five months after initial treatment when an oral carcinoma developed at the same site. She was 82 year-old, an ex-smoker, with a non-homogeneous verrucous type leukoplakia on the buccal mucosa excised with QMR scalpel. Initial histological report confirmed OL with mild dysplasia.

## Discussion

Due to the potential risk of malignant transformation of OL, the necessity for treatment is recommended by several authors (8). Mehana *et al.* (8) in a systematic review of 14 studies reported that surgery may reduce malignant transformation of OL with dysplasia, though it does not eliminate this risk completely. Many modalities of surgical treatments have been suggested including the use of new technologies such as the lasers. However, studies failed to demonstrate evidence of the advantage of the one treatment modality over another. This could be related to the fact that the majority of the studies analysing the efficacy of these treatment modalities are case-series studies most of them without comparison with traditional surgery (9).

Our data must be interpreted in the context of a retrospective study and taking into account other limitations such as the small number of patients specially divided to each treatment group, and the short follow-up time in some cases. Moreover, considering the retrospective nature of the sample, the subdivision of the lesions into different groups according to the type of treatment does not follow a random sequence. We ruled out all those lesions incorrectly diagnosed as OL. Also we compared various lasers with different wavelengths used in specialized laser units by experienced professionals with a traditional treatment control group. To the best of our knowledge this is the first analysis of the clinical outcome of OL treated with CO2 laser, Er:YAG laser, Nd:YAG laser, or QMR scalpel with a comparison group treated with traditional scalpel excision.

Local recurrences after surgical treatment, including laser treatments, are not uncommon (4). The overall recurrence rate of OL (taking into account all treatment groups) was 27.6%, which is within the range of 2 to 40% reported in the literature (13,14), and similar to that reported by Ishii *et al.* (10) (29.3%) in 154 lesions treated with CO2 laser, Nd:YAG laser or KTP laser or by Chandu and Smith (15) (28.9%) in 73 white lesions treated with CO2 laser. Different definitions for recurrence may result in different rates reported in the literature (14,16,17).

In the present study, recurrence was analyzed according to several socio-demographic variables such as gender, age and smoking/alcohol consumption. These, were not related to the clinical outcome and this aspect is consistent with other reports (14,16,18). Nevertheless, other studies reported higher recurrences rates in smokers (19,20), and alcohol consumers (15). We advised all patients to eliminate tobacco and alcohol habits before the beginning of treatment and during the follow-up. Perhaps if many patients maintained their habits the recurrence could be higher as tobacco and alcohol are etiological factors for OL.

The clinical-pathological variables including size, distribution, clinical type of OL, and the presence of dysplasia did not influence the presence of recurrence of the lesions studied here. Our findings are in agreement with few other previously reported (12,14,19). However, other studies that brought up factors that predicted the surgical outcome are noteworthy. For example, Chiesa *et al.* (21) observed that size of operated lesions was predictive of recurrence in 167 OL treated with CO2 laser. Yang *et al.* (20) reported the positive influence of CO2 laser surgery for treatment of 114 widespread dysplastic multiple-focus lesions on Taiwanese patients. Schoelch *et al.* (22) observed a high recurrence rate in patients with verrucous OL in a follow-up study of 70 patients submitted to CO2 laser or Nd:YAG surgery. Jerjes *et al.* (17) observed 19.5% recurrences that were related with the grade of dysplasia in 123 oral dysplastic lesions. These conclusions may be influenced by the subjectivity of interpretation of epithelial dysplasia in the grading system. Nevertheless, these clinical-morphological variables are considered as important determinants in the risk of malignant transformation of OL (1).

In the present study, there was one patient (1.5%) who developed an oral cancer, a rate that is consistent with the results of van der Hem *et al.* (12) and Ishii *et al.* (10). Interesting this was in a female patient, with a non-homogeneous OL, and with dysplasia at primary presentation. All three factors - female sex, non-homogeneous type and the presence of dysplasia – have been highlighted to be associated with higher rates of malignant transformation of OL (4). Nevertheless, the severity of dysplasia seems the most reliable predictor for malignant transformation to this date (23). Although, oral transformation of OL is one of the most important outcome of the treatment of these lesions, the small amount of patients (with only one case of OL transformation) did not allow us to analyse the existence of possible statistical differences between the treatment groups regarding OL transformation. The relationship between treatment modality of OL, recurrence and malignant transformation has been described in some studies with similar results (14,17). Moreover, recurrence of OL seems to be a prognostic indicator of oral malignant transformation (24). Involvement of more patients with longer follow-up in the present case series would be necessary in order to draw conclusive data on relationship between malignant transformation and type of treatment.

Regarding the different groups of surgical treatments of OL (traditional scalpel, QMR scalpel, Nd:YAG laser, Er:YAG laser, and CO2 laser), it was possible to observe that in every group of treatment there were some cases that recurred following treatment. Perhaps some cases of leukoplakias will recur independently of the type of treatment that they are submitted to, influenced by the genetic or epigenetic errors that individual cells could possess. We did not observe any significant differences between all treatment modalities groups based on a general analysis. This meant that the different wavelengths of lasers studied here are at least comparable with their ability to perform an excision procedure.

Laser technology offers advantages in both intra-operative and post-operative sessions: haemostatic effect of the laser light, fungicidal and bactericidal effect, improved healing of the surgical wound and possibility of healing by secondary intention, improved postoperative course with a reduction of pain, edema and infections (9,25). The use of lasers also allows the treatment of very extended or diffuse lesions (as for example in patients with PVL) for which surgical excision would not be practicable. On the other hand, if vaporization technique is used, this does not allow the histological analysis of the lesion.

The effectiveness of laser management of OL has been reported using CO2 laser (10,12,14,16-20,22,26), Nd:YAG laser (10,13,22,26,27), and KTP laser (10,11) with recurrence rates within 2% to 40% (13,14). However, in the present study, when we analyzed the clinical outcome comparing only each treatment group with the traditional scalpel group we observed a significant difference between the patients treated with the Er:YAG laser vs those treated with traditional scalpel excision. These results suggest that Er:YAG laser could be more effective in terms of clinical success when compared with the results obtained with the use of the traditional scalpel to treat OL (*P* = 0.015). Although, there are very few reports (with few patients) of the use of Er:YAG laser in the treatment of OL (27), this may be a very promising option in the treatment of OL. This particular laser has also the advantage of producing minimal histological artefacts compared to Nd:YAG laser or electrocautery, thereby allowing an efficient histological analysis of the excised tissue (27,28). The ability to perform microscopic examination of margins of a specimen is an important assessment to ensure complete excision (29). Knowing the exact thermal damage zone of biopsies related with different lasers or techniques is an important aspect to have in mind when these instruments are used and have been reported by several reports (28).

Although there were no differences in the effectiveness of the QMR scalpel with traditional scalpel, QMR scalpel has many advantages from the operational point of view: very rapid incision and with clean and regular cutting surfaces; absence of tissue carbonization; coagulation at temperatures not higher (63°C) and absence of substantial alterations of the tissues (30). Its application in the treatment of OL may be further warranted.

In conclusion we observed that the different lasers with varying wavelengths studied here were an efficient method to eliminate oral leukoplakia compared to traditional scalpel with the intrinsic advantages of lasers. Er:YAG laser may be an promising instrument to treat OL. Multicentric, randomized and controlled longitudinal studies are needed to better characterize the efficacy of these treatment modalities for OL. A program of long-term follow-up is indicated for all patients regardless of the therapeutic approach applied.
